# Influence of Online Merging Offline Method on University Students’ Active Learning Through Learning Satisfaction

**DOI:** 10.3389/fpsyg.2022.842322

**Published:** 2022-02-15

**Authors:** Huiju Yu, Shaofeng Wang, Jiaping Li, Gaojun Shi, Junfeng Yang

**Affiliations:** ^1^School of Marxism, Hangzhou Normal University, Hangzhou, China; ^2^School of Logistics and E-Commerce, Zhejiang Wanli University, Ningbo, China; ^3^Smart Learning Institute, Beijing Normal University, Beijing, China; ^4^Jing Hengyi School of Education, Hangzhou Normal University, Hangzhou, China

**Keywords:** OMO, online active learning, learning satisfaction, complaint, TAM, LS-SEM, MGA

## Abstract

Students’ active learning behavior determines learning performance. In post-COVID-19 period, Online Merging Offline (OMO) method become a common way of university students’ learning. However, at present, there are few studies in active learning behavior in the OMO mode. Combined with learning satisfaction and Technology Acceptance Model (TAM), this paper proposes an Online Active Learning (OAL) Model to predict the influencing factors of college students’ active learning behavior and then analyzes the differences between OMO model and pure online model by multi-group analysis (MGA) based on the model. The designed questionnaire was distributed, and a total of 498 valid questionnaires were collected. Using SmartPLS to analyze partial least squares structural equation modeling (PLS-SEM) and MGA, it is found that: (1) there are differences in the influencing factors of active learning between OMO and pure online model; the moderating effect of learning complaint in OMO mode is not established, and social isolation and age does not affect active learning in OMO mode; (2) learning quality, perceived ease of use, expectation, perceived usefulness, and social isolation indirectly affect active learning through learning satisfaction in both OMO model and pure online model; (3) learning satisfaction is an important mediating variable affecting active learning; and (4) learning complaints will negatively regulate the relationship between learning satisfaction and active learning only in pure online model. According to these findings, the paper provides theoretical and practical implementation suggestions implications for OMO teaching and OAL to ensure the expected learning outcome.

## Introduction

The COVID-19 has brought about great changes to education. Compared with the traditional classroom face-to-face teaching, more and more schools organize and carry out teaching in Online Merging Offline (OMO) mode (Huang et al., 2021), and students have gradually shifted from classroom learning to the mixed mode of offline and online learning. The development of distance online education shows that students’ online learning is prone to emotional loneliness, learning burnout, and a high drop-out rate (Kwon et al., 2010). Therefore, moderate face-to-face teaching has always been a basic method to improve the effect of distance learning (Flanagan et al., 2000). However, after the outbreak of the COVID-19, large-scale schools suddenly closed and transferred to online teaching. Students’ online learning in this situation has different characteristics from online learning in traditional distance education: (1) teachers and students in the class are familiar with each other; (2) teaching is usually organized in the form of administrative class; (3) the information literacy of teachers and students is not competent for distance education (Aydin and Erol, 2021), (4) inadequate teaching tools and platforms; and (5) insufficient learning resources (Huang et al., 2020). Online active learning (OAL) is a learning activity in which students actively discuss, summarize, and practice in the process of online learning (Christie and de Graaff, 2017). Therefore, exploring the factors affecting students’ online active learning, so as to improve students’ learning effect and learning satisfaction under the COVID-19, has become an important topic in the field of distance learning in the post-COVID-19 era.

Before the COVID-19, online learning research mainly focused on user attitude (Nayernia, 2020), online learning motivation (Chen and Jang, 2010), continuous online learning (Wu and Chen, 2017), etc. Since the outbreak of the COVID-19, online learning research has mainly focused on the role of technology (Singhal et al., 2021), online teaching methods and strategies (Zayapragassarazan, 2020; Orlov et al., 2021), learning experience and learning effect (Nguyen et al., 2021), etc. Since the outbreak of the COVID-19, under the situation of insufficient overall educational preparation and changes in classroom characteristics, there are few studies on the performance of students’ online active learning.

After the COVID-19 situation improved, the school reopened, teachers and students returned to school, and the OMO teaching mode was gradually accepted by most teachers (Huang et al., 2021) OMO mode advocates student-centered learning, where students’ learning space expands from physical classroom to online classroom, and provides students with flexible learning resources and methods, supporting students’ personalized learning (Yang et al., 2019, 2021). Compared with the lack of preparation at the beginning of the COVID-19, the characteristics of students’ online active learning in the OMO mode should be different from the online mode. The OMO model is of great significance to education development in the post-COVID-19 era. Therefore, what are the factors affecting online active learning in pure online mode and OMO mode should be investigated, so this paper intends to investigate the following questions:

RQ1: What are the core factors affecting students’ online active learning, and what is the internal structure and relationship between these core factors?

RQ2: What are the differences of the influencing factors between OMO mode and online mode?

To answer the research questions, this paper will first build a conceptual model of online active learning from the perspective of learners’ online learning behavior, based on the relevant theories of TAM and learning satisfaction. The next section of the paper will show the literature related to online active learning, TAM, learning satisfaction, and OMO, which is followed by the hypotheses for the study based on the literature. Then the section followed shows the study process, the instruments, and data analysis methods. The results are presented, and the differences of influencing factors of active learning between OMO mode and pure online mode are discussed, and guidance for OMO teaching in the post-COVID-19 era are also proposed. The paper finally concludes the strategies to promote active learning by OMO mode and an outlook toward future research directions.

## Literature Review and Hypotheses

The implementation of online active learning is an important factor affecting students’ learning success (Wang and Huang, 2020). Student-centered teaching design and teaching philosophy (Qureshi et al., 2016) help to promote students’ online active learning. In addition, a clear curriculum structure and necessary assistance can also promote college students’ active learning (Hung et al., 2010). The active learning process can make it easier for students to digest, understand, and master the knowledge points learned (Prince, 2004). Jamaludin and Osman (2014) found that emotional participation and action participation were the two most important types affecting active learning. From the perspective of learning results, active learning will significantly improve the learning effect (Hartikainen et al., 2019). Developing online learning systems suitable for students can stimulate students’ enthusiasm for active learning and bring better learning results (Hwang et al., 2019). Based on the research results of previous scholars, this paper defines online active learning as the learning behavior actively implemented by college students when participating in online learning (Wang et al., 2021a), which could be influenced by the following factors.

### Learning Satisfaction and Learning Quality

Learning Satisfaction (LS) is a subjective judgment between learners’ gains and expectations in the learning process (Wu et al., 2010), which is also considered as an important factor for learning behavior (Kim and Kim, 2016; Osama et al., 2019), learning success (Caskurlu et al., 2020), and learning experience (Huang, 2021). Sun et al. (2008) found that students’ coaches, courses, technology, architecture, and environment affect learners’ perceived satisfaction in online learning. Learning satisfaction is an important factor to promote learners to adopt learning behavior (Mohammadi, 2015). Interactions among learners, teachers, and learning materials positively impact learning satisfaction, and learning satisfaction will also affect learning motivation and learning effect (Lovecchio et al., 2015). The improvement of teaching methods can improve learners’ satisfaction, learning effect, and learning enthusiasm (Lee, 2008). Wu et al. (2010) concluded that the key factors affecting learning satisfaction in the blended learning environment include performance expectation, interaction, and learning environment. In flipped learning, Kim and Kim (2016) subdivided satisfaction into system convenience satisfaction, interaction satisfaction, support service satisfaction, and learning satisfaction to help teachers and researchers have an in-depth insight into the internal composition of satisfaction. Huong et al. pointed out that teachers, facilities, teaching materials, and learning environment have a positive impact on students’ learning satisfaction (Huong et al., 2017). Osama et al. (2019) explored task technology suitability (TTF) and compatibility based on the integration of TAM and DMISM models and confirmed that overall quality and compatibility are important factors affecting learning satisfaction. Learning satisfaction helps students obtain good learning results (Caskurlu et al., 2020). In this study, learning satisfaction is defined as learners’ perceived gain from online learning (Nagy, 2018). Based on the above analysis, the hypothesis proposed in this paper is:

*H1:* Learning satisfaction has a positive impact on online active learning.

Learning Quality (LQ) refers to the extent to which the quality of the learning systems, learning service, and learning content meet the needs of learners (Lin et al., 2020). The stability, fluency, accessibility, and visual design of the system have become the key factors affecting users’ use of the online system (Molla and Licker, 2001), while the unfriendly system experience will hinder users’ continued use of the system (DeLone and McLean, 2004). The functional perfection and user experience of online learning system will further affect college students’ online active learning attitude, willingness, and behavior (Lin, 2007). The research results of Almaiah et al. (2016) showed that the qualities of learning content, content design, functionality, user interface design, accessibility, personalization, and responsiveness are the antecedents of accepting mobile learning. Kadam (2020) believed that an appropriate learning system played an important role in improving learning effects. Tao et al. (2019) confirmed in the research on Massive Open Online Courses that learners’ perceived quality had a strong indirect impact on behavioral intention. Online learning pays more attention to learning quality than traditional learning (González-Gómez et al., 2012). As a result, the overall quality of online learning is strongly linked to learning satisfaction (Hong et al., 2016). Therefore, this study introduces system quality into the research model. Based on the above analysis, the hypothesis proposed in this study is:

*H2:* Learning quality has a positive impact on learning satisfaction.

### Technology Acceptance Model

TAM has been a common basic theory in online learning research (Wang and Huang, 2020; Wang et al., 2021b). TAM can explain the causes of online learning behavior (Wang et al., 2021a). Two specific variables perceived usefulness and perceived ease of use are considered the decisive factors affecting behavior in the online learning environment (Alfadda and Mahdi, 2021). Combining external factors, TAM can improve the prediction effectiveness of the model (Wang, 2020; Unal and Uzun, 2021). Perceived satisfaction can not only predict learning performance and tendency of continuous learning to a great extent, but also promote cooperation and sharing among learners, so as to effectively improve the effect of online learning (Dai et al., 2017). The teaching quality of the online learning environment positively impact students’ acceptance (Larmuseau et al., 2019). Based on TAM model, Guo et al. (2016) found that students’ online learning persistence intention is significantly affected by their perceived usefulness, enjoyment, and satisfaction. Liu et al. (2010) discussed the factors affecting online learning intention, including online course design, user interface design, and previous learning experience. Choudhury and Pattnaik (2020) reviewed the previous successful research results of online education and found that learners’ ease of use of technology and perceived usefulness of online courses determine the effectiveness of learners’ online learning. Learners’ perceived usefulness and ease of use will increase learning satisfaction, and perceived usefulness and satisfaction in learning will create positive use intention (Granić and Marangunić, 2019). The research of Shin and Kang (2015) confirmed that students’ perceived ease of use and perceived usefulness would affect learning satisfaction through mediation variables (such as behavioral intention) when they conducted mobile learning. Therefore, this study will build a research model of online active learning based on TAM. Based on the above analysis, the hypothesis proposed in this paper is:

*H3:* Perceived ease of use has a positive impact on learning satisfaction.

*H4:* Perceived usefulness has a positive impact on learning satisfaction.

### Social Isolation and Expectation

Rubin et al. (1993) defined social isolation (SI) as a lack of social interaction and rejection or isolation by peer groups. Raza et al. (2021) pointed out that social isolation is a key factor affecting Pakistani students’ use of learning management systems. Bester and Budhal (2001) found that learners’ academic performance is related to social isolation. Learners with good performance tend to be more confident and have higher social status among peers; learners with poor academic performance are at greater risk of becoming social isolators. Social isolation is the separation of physical space and the result of lack of interaction and communication (Modarresi Yazdi, 2014). Social isolation reduces the links between individuals and between individuals and groups (Falahi et al., 2020), with many negative effects, such as negative impacts on students’ online active learning and online active learning willingness (Wang and Huang, 2020). As a measure to avoid the influence of the COVID-19, social isolation has greatly affected the work of teachers and the learning process of students. Teachers and students need innovative teaching and learning methods to reduce the negative effects of social isolation (Scavarda et al., 2021). Based on the above analysis, this study defines the sense of social isolation as the isolation and separation felt by learners when they carry out online learning (Rasheed et al., 2019) and puts forward the hypothesis:

*H5:* Social isolation has a negative impact on learning satisfaction.

Parasuraman et al. (1988) defines user expectations as the user’s perception and feeling of a product or service. Expectation is an important predictor of academic achievement (Reeve and Tseng, 2021), when students’ expectations are met or exceeded, a higher level of satisfaction will appear (Lin et al., 2020). Wu et al. (2010) pointed out that performance expectations contributed the most to learning satisfaction. Therefore, according to the above analysis, the hypothesis is proposed:

*H6:* Expectation has a positive impact on learning satisfaction.

As mentioned in the previous part, learning satisfaction in this study is learners’ perceived gain from online learning. Previous research also showed that learning satisfaction acted as the mediating variable to influence the active learning. The research of flipped classroom indicated that learning satisfaction had a partial mediating effect on learning effectiveness (Lin and Chen, 2016). In the context of COVID-19 distance learning, learning satisfaction mediated between learning flow and learning outcomes (Kim and Park, 2021). Therefore, based on the multiple roles of satisfaction as a mediator variable in learning, the hypothesis is proposed:

*H7:* Learning satisfaction mediates the relationship between (a) perceived ease of use, (b) perceived usefulness, (c) social isolation, (d) expectation, and (e) learning quality.

### The Moderating Role of Learning Complaint

A customer complaint is an important antecedent affecting customer satisfaction and loyalty (Fornell et al., 1996), and a customer complaint is a form for consumers to express dissatisfaction (Maxham and Netemeyer, 2002). In this study, learning complaints are defined as students’ complaints about dissatisfied aspects during online learning. In the learning environment, some studies have found a close relationship between learning complaints and satisfaction or dissatisfaction (Michalos and Orlando, 2006). Lin et al. (2020) found that the more satisfied international students are with the school’s educational services, the fewer their complaints and the more positive their views on the school’s handling of student complaints. It is assumed that learning complaints have a moderating role on learning satisfaction and active learning, that is, the learning complaints will reduce the relation of satisfaction and active learning. Therefore, the hypothesis proposed in this study is:

*H8:* Learning complaint has a negative moderating effect on the relationship between learning satisfaction and active learning.

### Online Merging Offline Education

OMO refers to providing learners with online and offline learning space to meet their learning needs anytime and anywhere (Huang et al., 2021). It is an important form of education development in the future (Xiao et al., 2019). Institutional and resource support, home learning atmosphere, and the sense of learning presence of teachers and students affect learning satisfaction and subsequent continuous learning behavior in the OMO environment (Wang et al., 2021c). With the application of emerging intelligent technologies, the OMO mode has developed rapidly, which provides great convenience for promoting active learning (Han and Ellis, 2019). Learners can use the online digital learning resources to carry out active learning, and teachers have changed from the role of teaching to the role of supporters and coaches (Ashraf et al., 2021). This student-centered blended learning concept has been widely used in online teaching during the COVID-19 (Mansoori et al., 2020; Huang et al., 2021; Yunus et al., 2021). Mansoori et al. (2020) found that the satisfaction and learning effect in OMO mode are better than face-to-face learning. OMO method has a significant effect on promoting students’ active learning (Shimizu et al., 2019). Therefore, this study will introduce OMO as a group variable to explore the differences in active learning behaviors in OMO and pure online mode. Based on the above analysis, the hypothesis proposed in this study is:

*H9:* The influencing factors of active learning behaviors between OMO students and pure online students are different.

### Control Variables

Research in e-learning usually concluded that male students were more willing to use and learn computers than female students (Li and Kirkup, 2007), and male students had a more positive perception of e-learning than female students (Ong and Lai, 2006). Lu and Chiou (2010) found that male students have higher evaluation and satisfaction with e-learning than female students. Different from the above conclusion, González-gómez et al. (2012) indicated that female students are more satisfied with e-learning subjects than male students. Some studies have found that students’ age also has a significant impact on their online learning satisfaction or academic performance (Dabbagh, 2007). Lim et al. (2006) pointed out that the younger (20–29 years) learners performed significantly better in knowledge tests and were more satisfied with the quality of online courses. Ke and Kwak (2013) examined whether online learning interactive participation, perception, and learning satisfaction are consistent among different ages. The analysis results of online discussion records showed that age cannot predict the quantity and quality of students’ posts. While in Wang’s studies (Wang et al., 2021a), older adult students were often reluctant to take online active learning. Therefore, gender and age were added to the research model as control variables.

Based on the above analysis, the assumptions proposed in this paper are:

*H10:* Gender has impacts on online active learning.

*H11:* Age has impacts on online active learning.

### Conceptual Model

**Figure 1:**
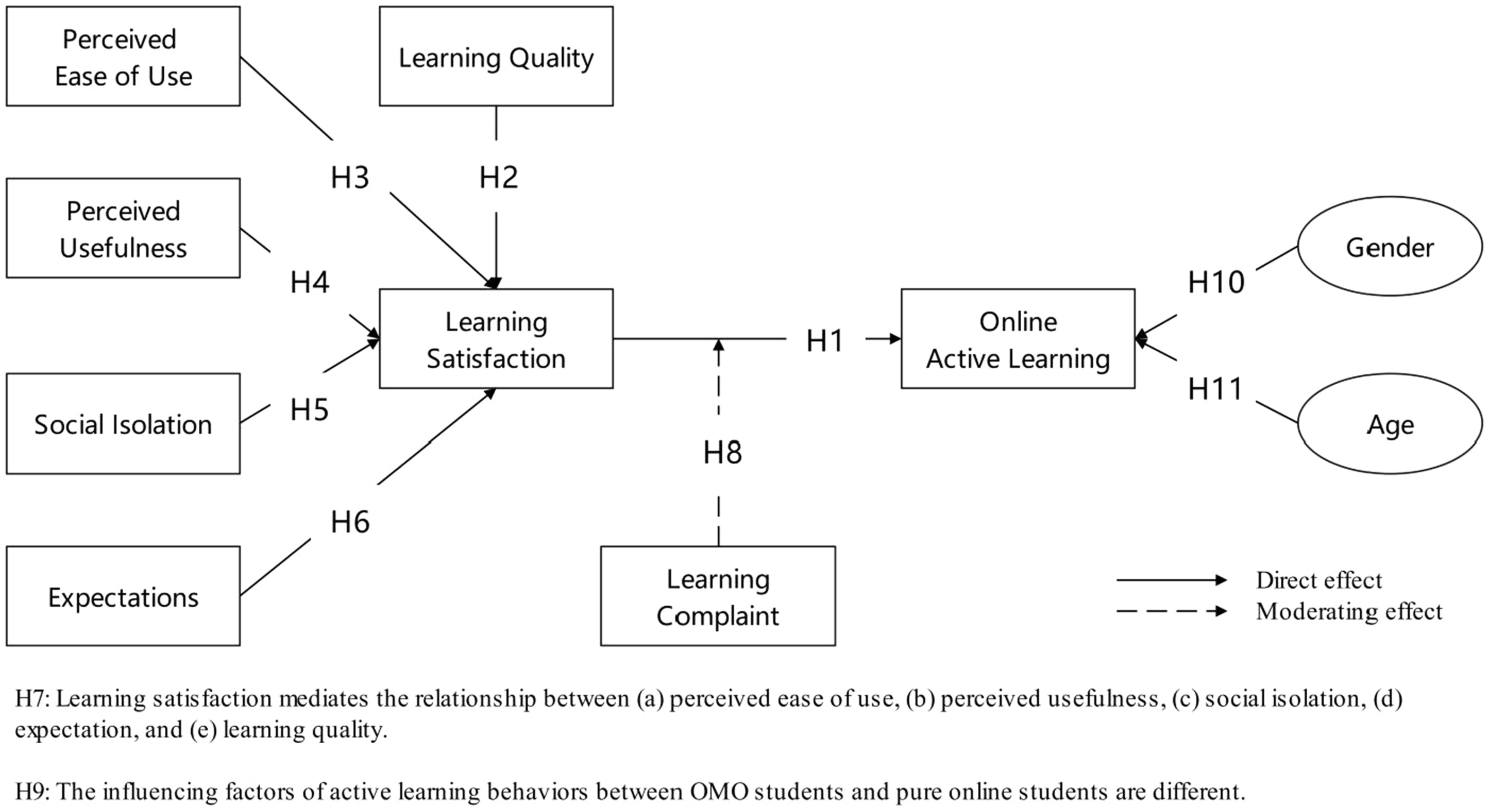
Conceptual model.

Supported by learning satisfaction theory and Technology Acceptance Model (TAM), this study constructs a conceptual model of online active learning (see Figure 1), to explore the effects of social isolation, expectation, learning quality, perceived ease of use, and perceived usefulness on learning satisfaction, and also to test the mediating effect of learning satisfaction and the moderating effect of learning complaint, and also to compare the differences of these factors in OMO mode and online mode.

## Research Methods

Due to the research model proposed in this study containing latent variables that cannot be observed directly, so the research data will be collected by questionnaire after developing the measurement scale (Prasad et al., 2018; Osama et al., 2019; Wang and Huang, 2020).

### Instruments

To ensure the content’s effectiveness, all construct items were from or adapted from existing literature (Hair et al., 2016; Wang, 2020). The measurement items of perceived usefulness and perceived ease of use are from Wang et al. (2021b); the measurement items of learning satisfaction are from Mohammadi (2015) and Osama et al. (2019). The measurement items of social isolation are from Garrison et al. (2010) and Wang and Huang (2020); the measurement items of expectations are from Hossain and Quaddus (2012) and Prasad et al. (2018); the measurement items of learning complaint are from Wang et al. (2021a); the measurement items of online active learning are from Venkatesh et al. (2003) and Wang and Huang (2020); the measurement items of learning quality come from Vernadakis et al. (2011) and Osama et al. (2019); each construct has 3 to 5 measurement items, and the 5-point Likert scale (Prasad et al., 2018) is used to reflect strongly disagree (1) to strongly agree (5).

After the preliminary design of the questionnaire, the WJX (an online questionnaire platform) was used to publish the questionnaire online. 30 students participated in the presurvey. After collecting the feedback information in the presurvey process, the questionnaire was optimized (Xu and Du, 2018; Huang et al., 2021). The formal questionnaire consists of two parts. The first part is the basic information (gender and age), and the second part is the measurement of 8 latent variables in the online active learning model (in Table 1).

**Table 1:** Instrument.

Construct	Items
Learning complaint	1.If learning process are not satisfactory, I will have the idea of complaining. 2.If learning process are not satisfactory, I will complain to my classmates. 3.If learning process are not satisfactory, I will complain to the teacher. 4.If learning process are not satisfactory, I will post a comment through the network.
Expectation	1.The experience of Active learning is better than I expected. 2.The service of Active learning is better than I expect. 3.The effect of Active learning is better than I expected. 4.In short, the results of Active learning are better than I expected.
Learning Quality	1.Learning support system to ensure my learning effect. 2.Learning support system provides collaborative learning. 3.Learning support system provides the necessary functions, such as questions and discussions. 4.Active learning provides the possibility of communication with other students. 5.Active learning appropriate with my learning style.
Learning Satisfaction	1.Active learning is enjoyable. 2.Active learning satisfies my learning needs. 3.Active learning makes me more confident. 4.I am satisfied with the Active learning process.
Active learning	1.I actively participate in the discussion of online learning. 2.I actively summarize the knowledge learned after class. 3.I am pleasure to practice the knowledge learned online. 4.I conduct Active learning frequently. 5.I often visit the Active learning system.
Perceived ease of use	1.Active learning is easy to carry out. 2.Active learning is easy to learn. 3.Active learning is convenient to carry out.
Perceived usefulness	1.Active learning can improve my learning efficiency. 2.Active learning can improve my performance. 3.Active learning can help me accomplish my learning goals. 4.Active learning is effective.
Social isolation	1.Active learning reduces the opportunities for communication between students and students. 2.Active learning reduces the opportunity for communication between students and teachers. 3.Active learning reduces discussion between students and students and teachers. 4.Active learning has given me a sense of isolation.

### Participants

The online questionnaire was used for the survey, and students from 4 Chinese universities and about 16 classes took part in the survey. Universities teachers invited students to participate in the survey through QR code during recess. The invited students can choose whether to participate in the survey according to their own wishes and have been informed the survey’s purpose. The online questionnaire was launched and collected for a month, and 498 valid questionnaires were obtained after data cleaning. Among the samples, 224 were male, accounting for 45%; 274 were female, accounting for 55%; 14 were aged 17 and below, accounting for 2.8%; 235 were aged 18–29, accounting for 47.2%; 124 were aged 21–23, accounting for 24.9%; 102 were aged 24–26, accounting for 20.5%; more than the number of people equal to 27 years old is 23, accounting for 4.6%. Among the respondents, 223 people were participating in OMO education, accounting for 44.8%; 275 people experienced online education during COVID 19 for more than 3 months, accounting for 55.2%.

## Results

Compared with other analysis technologies, partial least squares structural equation modeling (PLS-SEM) is suitable for developing new theoretical models, exploring complex models, for prediction purposes, and conducting exploratory research (Hair et al., 2016; Prasad et al., 2018). Therefore, this study used PLS-SEM analysis software SmartPLS 3 to test the model and verify the hypothesis (Sarstedt and Hwa, 2019).

### Measurements Model

Content validity, discriminant validity, and convergent validity are used to test the validity and reliability of the study model. All measurement items in the questionnaire are from literature and have been verified by presurvey before, so they are considered to have acceptable content validity (Osama et al., 2019). The results of the average variance extracted (AVE) are greater than 0.5 (in Table 2); the square root of AVE is greater than the correlation coefficient between the relevant variable and other variables (in Table 3). The above results show that the model has good convergent validity and discriminant validity (Wang et al., 2021c). Cronbach’s alpha and composite reliability (CR) are greater than 0.8 (in Table 1), indicating that the measurement model has good reliability.

**Table 2:** AVE, CR, and Cronbach’s alpha.

Construct	Coding	Item	Cronbach’s alpha	CR	AVE
Learning complaint	CO	5	0.851	0.894	0.627
Expectation	EPC	4	0.820	0.881	0.650
Learning Quality	LQ	4	0.824	0.883	0.655
Learning Satisfaction	LS	4	0.846	0.896	0.684
Active learning	AL	5	0.835	0.884	0.603
Perceived ease of use	PEU	3	0.830	0.898	0.746
Perceived usefulness	PU	4	0.855	0.902	0.696
Social isolation	SOI	5	0.883	0.915	0.682

**Table 3:** Correlation coefficient between latent variables and square root of AVE.

	AL	CO	EPC	LQ	PEU	PU	SAT	SOI
AL	**0.777**							
CO	−0.265	**0.792**						
LE	0.332	0.073	**0.806**					
LQ	0.346	0.061	0.274	**0.809**				
PEU	0.341	0.107	0.251	0.276	**0.864**			
PU	0.331	0.008	0.197	0.279	0.227	**0.834**		
SAT	0.588	−0.141	0.431	0.46	0.445	0.394	**0.827**	
SOI	−0.232	−0.006	−0.155	−0.127	−0.149	−0.115	−0.315	**0.826**

The value bold on the diagonal is the square root of AVE.

The analysis results show that the measurement model has good convergence validity and discriminant validity, because the factor loading between each measured variable and its latent variable is greater than the cross factor loading between other latent variables (Wang et al., 2021c). The results of cross-loadings show that the external load of each construct is greater than the cross load of other constructs. In addition, the maximum heterotrait–monotrait ratio (HTMT) of the correlations is 0.697 (<0.9; in Table 4). The above results indicate that the measurement model has good discriminant validity (Hair et al., 2016).

**Table 4:** HTMT.

	AL	CO	LE	LQ	PEOU	PU	SAT	SOI
AL								
CO	0.312							
LE	0.398	0.093						
LQ	0.416	0.089	0.331					
PEOU	0.407	0.128	0.304	0.333				
PU	0.391	0.043	0.237	0.331	0.269			
SAT	0.697	0.163	0.516	0.551	0.529	0.461		
SOI	0.268	0.034	0.178	0.151	0.169	0.130	0.363	

### Common Method Bias

The data are from the questionnaire survey, and there is the possibility of common method bias (CMB; Podsakoff et al., 2003). In this study, Harman’s single factor test was used to detect the existence of CMB. SPSS 25 analysis showed that the variance interpretation of the first factor was 21.013% of the total variance which was less than 40%, and no factor could explain most of the variance. Therefore, we believe that CMB do not have a significant impact on this study (Lindell and Whitney, 2001).

### Structural Model

**Figure 2:**
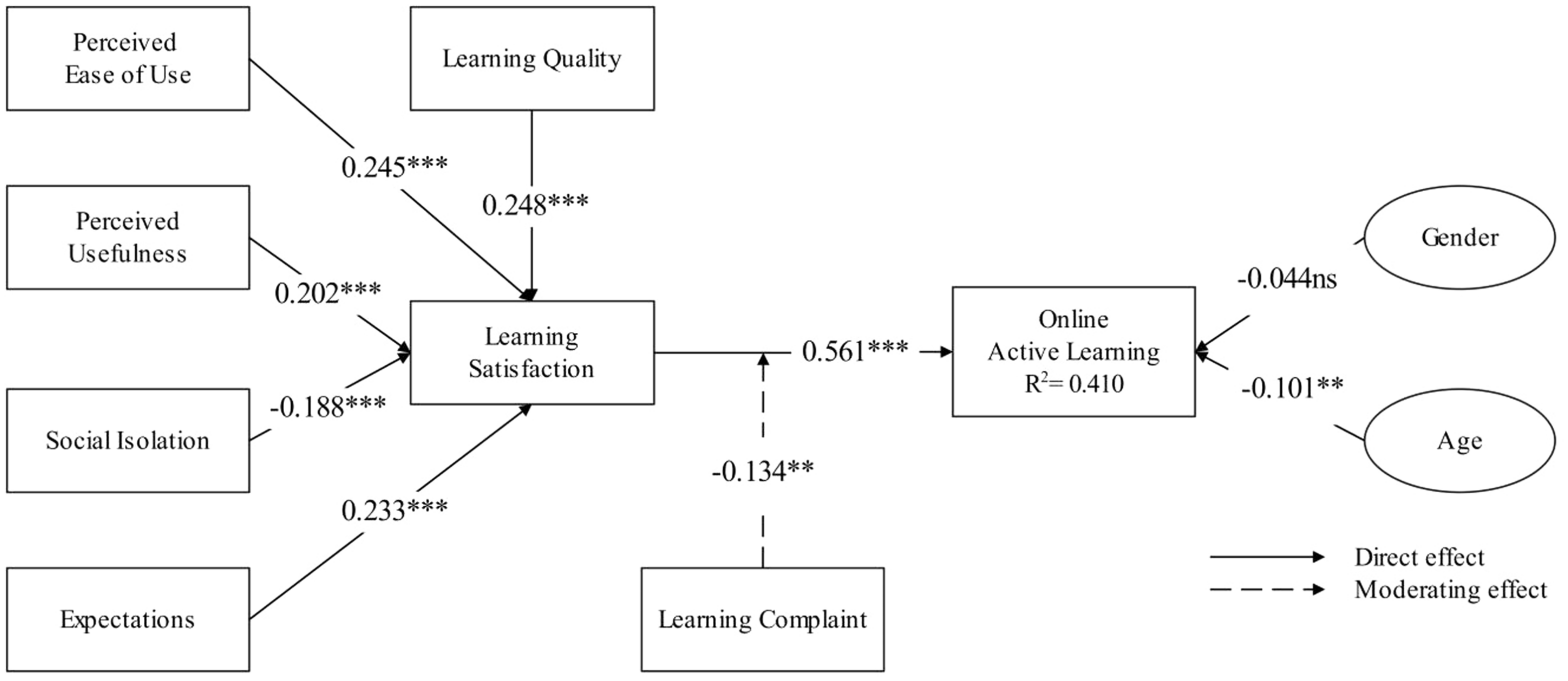
PLS results of online active learning model. ^**^p < 0.01 and ^***^p < 0.001. ns, not-significant.

We analyzed the structural model with the help of SmartPLS 3 (Hair et al., 2016). The R2 of learning satisfaction and online active learning are 0.462 and 0.410, respectively (see Figure 2). The prediction effect of the online active learning model is good (Wang et al., 2021c). The Q2 values of learning satisfaction and online active learning are 0.312 and 0.240, respectively, indicating that the online active learning model has a medium prediction effect. The standardized root mean square residual (SRMR) is an important indicator to measure the fit criteria of the PLS-SEM model. The SRMR of the proposed model is 0.042 (<0.05), indicating that the model has a good degree of fit (Hair et al., 2016).

### Mediation and Moderation

This study uses Bootstrapping to test the mediating influence of learning satisfaction, as shown in Table 5.

**Table 5:** Mediation results.

Path	Influence	95% Confidence intervals	Value of *P*	Significance	Mediation
PEU → LS → OAL	Direct effect	(0.048, 0.196)	0.000	Yes	Partial
	Indirect effect	(0.050, 0.117)	0.000	Yes	
PU → LS → OAL	Direct effect	(0.034, 0.185)	0.000	Yes	Partial
	Indirect effect	(0.040, 0.097)	0.000	Yes	
SOI → LS → OAL	Direct effect	(−0.149, 0.009)	0.001	Yes	Partial
	Indirect effect	(−0.092, 0.036)	0.001	Yes	
EPC → LS → OAL	Direct effect	(0.044, 0.184)	0.000	Yes	Partial
	Indirect effect	(0.048, 0.111)	0.000	Yes	
LQ → LS → OAL	Direct effect	(0.027, 0.179)	0.000	Yes	Partial
	Indirect effect	(0.051, 0.117)	0.000	Yes	

According to the analysis method of mediation effect recommended by Zhao et al. (2010), it is found that the relationships between perceived ease of use (PEU), social isolation (SOI), perceived usefulness (PU), Expectation (EPC), learning quality (LQ), and online active learning (OAL) are partially mediated by learning satisfaction (LS; in Table 3).

Using the test of the moderating effect of SmartPLS 3 on learning complaint (Hair et al., 2016), the analysis results show that the moderating variable learning complaint has a negative moderating effect on the relationship between learning satisfaction and online active learning (see Figure 2), and the research hypothesis is verified (Hair et al., 2016).

### Multi-Group Analysis

Partial least squares multi-group analysis (PLS-MGA) can help researchers understand the differences in influence relations caused by specific grouping variables (Hair et al., 2016). This study first divides the sample data into two groups of OMO model and pure online education model, and then uses SmartPLS 3’s multi-group analysis (MGA). The results of function calculation are shown in Table 6.

**Table 6:** PLS-MGA results.

	Path coefficient		Value of *P*	
	OMO model	Online education model	OMO model	Online education model
EPC → SAT	0.274	0.203	0.000	0.000
LQ → SAT	0.312	0.178	0.000	0.000
PEU → SAT	0.148	0.347	0.006	0.000
PU → SAT	0.220	0.174	0.000	0.000
SAT → OAL	0.538	0.585	0.000	0.000
SOI → SAT	−0.091	−0.266	0.059	0.000
Moderation of CO	−0.080	−0.229	0.418	0.013
Age → OAL	−0.064	−0.124	0.230	0.005
Gender → OAL	−0.027	−0.060	0.620	0.168

In the group of students who adopt the OMO method, the moderating effect of CO is not significance (*β* = −0.080, *p* > 0.4), SOI (*β* = −0.091, *p* > 0.05) did not significantly affect SAT, Age (*β* = −0.064, *p* > 0.23) had no impact on OAL. Regardless of whether OMO or online education teaching methods were adopted, SAT positively affected OAL; PU, PEU, EPC, and LQ positively affected SAT; Gender did not affect OAL.

This study tested all the hypotheses through structural equation model and multi-group analysis. Except H9 hypothesis was not supported, other research hypotheses were verified.

## Discussions

### OMO Model and Pure Online Model

The multi-group analysis on the OMO model and pure online model show that learning satisfaction has a significant positive impact on online active learning whether using OMO teaching or not. In addition, the factors that have a significant positive predictive effect on learning satisfaction are perceived usefulness, expectation, and learning quality. However, in the grouping model with OMO mode, it is found that social isolation, learning complaint, and age are not factors that affect active learning, while the above three assumptions in the pure online model are still important factors that affects active learning. The reason for the above results lied in that individuals mainly complete learning activities alone in pure online model, and communication and cooperation with peers are also completed online. On the one hand, the cyber space is easy to causes a sense of isolation and brings lonely feelings to learners. Once learners find that the online learning process is not satisfactory, it is easy to cause learning complaints. Social isolation and learning complaints will affect learning satisfaction and therefore online active learning. On the other hand, with pure online model, students will feel that the online learning system is not easy to use if the system is not designed perfectly for learning, which will reduce learners’ satisfaction and impede online active learning.

Correspondingly, with OMO model, the offline face-to-face teaching can help students resolve some learning complaints and social isolation in the online learning process. At the same time, teachers can also explain and help the technical and learning problems in the online learning process. In fact, with OMO model, the advantages of convenience, flexibility, and personalization for online teaching and the advantages of experiencing, feeling, and sense of presence for offline teaching are combined together to provide efficient and effective learning. The disadvantages of social isolation (sense of loneliness) and complaints for online teaching could be relieved by integrating suitable offline teaching. OMO provides the opportunities for both teachers and students for flexible and resilient teaching and learning.

### Perceived Usefulness and Perceived Ease of Use

Data analysis shows that perceived usefulness and perceived ease of use have impact on student’s active learning behavior whether in OMO model or pure online model, which shows consistence with the previous research in distance learning (Kim et al., 2021). In order to promote the perceived usefulness and ease of use in OMO mode, not only the technology features should be considered, but also the environment, the learning contents, the learning path, and the learning assessment should be redesigned for online and offline learning scenarios. Teachers should organize and enrich the teaching content not only for the objective of knowing and understanding, but also for the higher cognitive learning objectives of evaluating and creating. The intelligent features of learning management systems could provide students the personalized learning resources, learning pace, and even assessment according to students’ learning traits. How to ensure the seamless infusion of online and offline learning is the key for teachers who utilize OMO mode. The advantages of online and offline learning should be promoted, while the disadvantage of both online and offline learning should be overcome: give play to the complementary role of online and offline learning, establish relevant mechanisms for teachers’ online and offline guidance, and enhance the support for students’ online active learning.

### Social Isolation and Expectation

This sense of social isolation may come from two aspects: one is the isolation of the social environment caused by the outbreak of the COVID-19, and the other is the isolation of the online virtual environment. The effectiveness of online learning relies on a good sense of social presence and interaction (Tu, 2002). Teachers should pay more attention to the form and organization of online teaching, and create an interactive, supportive and feedback online learning atmosphere to reduce the sense of isolation in the online virtual environment. OMO model can effectively make up for the lack of social presence and mutual movement brought by students’ online learning. In OMO mode, teachers should timely follow up students’ learning process and find problems, organize students to carry out cooperative learning and group cooperation, timely solve students’ personalized problems in the learning process, and give emotional support to effectively reduce students’ sense of online isolation.

According to the results of data analysis, expectation significantly affects learning satisfaction, which proves that expectation is also an important factor affecting learners’ online active learning, which is also similar to the existing research results (Prasad et al., 2018; Xu et al., 2018). Expectations are learners’ belief that they could successfully accomplish their learning objectives. When online learning can meet the expectations, learning satisfaction will increase and learners’ online active learning will be greatly improved; on the contrary, when expectations cannot be met, learning satisfaction will be low and learners’ online active learning will be greatly reduced. In OMO model, teachers could reduce the gap between curriculum objectives and expectations of students, by adjusting the instructional content to form the new objectives by considering most students’ expectations and the new OMO learning scenario.

### Learning Quality

Learning quality is learners’ subjective feeling and evaluation of the learning effect. The understanding of knowledge and the growth of ability are important indicators to evaluate the learning quality. Information technology, multimedia technology, 5G and other new generation technologies provide important carriers for learners to carry out online active learning (Han and Ellis, 2019; Ashraf et al., 2021), which is an important way to improve learners’ learning quality. To promote the learning quality in OMO mode, the reliable network infrastructure, the friendly tools and platforms, the quality digital learning resources, the teacher and students’ digital competency, and the suitable pedagogies should be considered in the initial planning stages. In implementing OMO, no matter online or offline, Showing (S) the learning content clearly, Managing (M) the classroom environment actively, Accessing (A) to learning resources easily, Reacting (R) with students timely, Tracking (T) the learning process precisely compose the “SMART” principle, which should be considered for promoting learning quality.

### Mediation of Learning Satisfaction

The results of data analysis show that learning satisfaction is a key factor in online active learning. Learning satisfaction mediates perceived ease of use, perceived usefulness, social isolation, expectation, learning quality, and its role in online active learning. Learning satisfaction involves expectation, learning process, learning results, individual emotional feelings, and other levels. It refers to learners’ subjective evaluation of knowledge mastery and ability development and points to the interaction between subjects, individual subjective feelings, and emotional states in the learning process. Therefore, to improve the learning satisfaction of online learners, teachers should pay attention to the subjective status of learners and take learners as the center. In addition, teachers should pay much attention to the interactions between subjects. For example, teachers and students should strengthen personalized answers and guidance, students should strengthen discussion and communication, family members should strengthen emotional communication, and give full play to the effective support role of multiple subjects for learners. By controlling the prefactors of learning satisfaction, we can improve learners’ learning satisfaction and promote the implementation and development of online active learning.

### Moderator of Complaint

This study uses learning complaints as a moderating variable for online active learning models. The moderating effect test results confirm that learning complaint has a negative moderating effect on the relationship between learning satisfaction and online active learning, indicating that when the level of learning complaint is higher, the impact of learning satisfaction on online active learning will also decline. Learning complaints often have a clear direction. The object of complaints often lies outside the learners themselves, and the specific content of complaints often does not meet the learners’ expectations. For example, learners will complain about unstable network signals, too slow network transmission speed, system jam, inability to get technical support, etc. Learning complaint has strong emotional color, which has a considerable negative impact on online active learning and learning satisfaction. This enlightens educators to pay attention to learners’ feedback and set up certain interactive spaces and feedback channels for communication between learners and education managers. On the one hand, learners can release and relieve learning complaints in time to reduce negative emotions and psychological pressure. On the other hand, let education managers know what is unsatisfactory in the current education process and make timely adjustments and repairs, to reduce learners’ learning complaints and improve learners’ satisfaction. Communication between learners is also an important measure to resolve learning complaints. Through mutual communication, learners find that other learners also encounter the same and similar problems, but these external factors have no substantive impact on the core part of problem-solving, which consciously reduces learning complaints, and may further enhance the implementation of online active learning and further find solutions to problems.

## Conclusion, Limitation and Future Study

Aiming at the emerging OMO teaching, this study explores the influencing factors of online active learning and compares the differences of influencing factors of online active learning between OMO teaching and online teaching. The samples are empirically analyzed by SmartPLS 3 analysis software, and the research hypothesis test is carried out with the help of partial least squares structural equation model, and an online active learning model is proposed. On this basis, this study explored the relationship between perceived usefulness, social isolation, ease of use, expectation and learning effect and online active learning, and analyzed the mediating effect of learning satisfaction and the moderating effect of learning complaint.

By comparing OMO teaching with online teaching, we find that simple online teaching methods can easily lead to students’ sense of social isolation and learning complaints, while OMO mixed teaching method can well solve these problems. Face-to-face emotional interaction between teachers and students, hand-in-hand guidance and help, and face-to-face communication and cooperation between students reduce students’ sense of social isolation. It reduces students’ learning complaints, enhances students’ ability to grasp technology and other problems and improves the implementation probability and effect of students’ online active learning.

Comparing with pure online mode, the OMO mode has the intrinsic advantages. To prepare the OMO mode, school and teachers should build the reliable environments, provide suitable digital tools and digital learning resources, change the assessment approach, and assist teachers and learners with necessary supports. However, the key is the pedagogical issue related to smart teaching. Whether online or offline, the teaching could follow the “SMART” principle to promote learning quality and meet expectation.

This study also has a certain limit, the sample size and representativeness may be one-sided, and the application of online active learning model can be further expanded. In the future, it is planned to further explore the OMO teaching model, combined with the design of teaching process, and according to the online learning model proposed in this study, to further explore the influencing factors of OMO learning effect.

## Data Availability Statement

The original contributions presented in the study are included in the article/supplementary material, further inquiries can be directed to the corresponding author.

## Author Contributions

HY, JY, and SW: conceptualization. SW: methodology and software. SW and JL: validation. HY: investigation. JY: resources, funding acquisition, and supervision. HY, JY, JL, and SW: writing—original draft preparation. HY, JY, GS, and SW: writing—review and editing. All authors have read and agreed to the published version of the manuscript.

## Funding

This research work was supported by 2021 Zhejiang Provincial Philosophy and Social Planning Project “The construction of cloud employment system for higher education institute in post-COVID-19 period” (No: 21GXSZ030YB).

## Conflict of Interest

The authors declare that the research was conducted in the absence of any commercial or financial relationships that could be construed as a potential conflict of interest.

## Publisher’s Note

All claims expressed in this article are solely those of the authors and do not necessarily represent those of their affiliated organizations, or those of the publisher, the editors and the reviewers. Any product that may be evaluated in this article, or claim that may be made by its manufacturer, is not guaranteed or endorsed by the publisher.
